# A Rapid Capillary-Pressure Driven Micro-Channel to Demonstrate Newtonian Fluid Behavior of Zebrafish Blood at High Shear Rates

**DOI:** 10.1038/s41598-017-02253-7

**Published:** 2017-05-16

**Authors:** Juhyun Lee, Tzu-Chieh Chou, Dongyang Kang, Hanul Kang, Junjie Chen, Kyung In Baek, Wei Wang, Yichen Ding, Dino Di Carlo, Yu-Chong Tai, Tzung K. Hsiai

**Affiliations:** 1 Department of Bioengineering, University of California Los Angeles, Los Angeles, CA 90095 USA; 2 Department of Medical Engineering, California Institute of Technology, Pasadena, CA 91125 USA; 3 Division of Cardiology, Veterans Affairs Greater Los Angeles Healthcare System, Los Angeles, California, 90073 USA; 4 Department of Electrical Engineering, Peking University, Beijing, 100871 China; 5 California NanoSystem Institute, University of California Los Angeles, Los Angeles, CA 90095 USA; 6 Department of Medicine (Cardiology), School of Medicine, University of California Los Angeles, Los Angeles, CA 90095 USA

## Abstract

Blood viscosity provides the rheological basis to elucidate shear stress underlying developmental cardiac mechanics and physiology. Zebrafish is a high throughput model for developmental biology, forward-genetics, and drug discovery. The micro-scale posed an experimental challenge to measure blood viscosity. To address this challenge, a microfluidic viscometer driven by surface tension was developed to reduce the sample volume required (3μL) for rapid (<2 min) and continuous viscosity measurement. By fitting the power-law fluid model to the travel distance of blood through the micro-channel as a function of time and channel configuration, the experimentally acquired blood viscosity was compared with a vacuum-driven capillary viscometer at high shear rates (>500 s^−1^), at which the power law exponent (n) of zebrafish blood was nearly 1 behaving as a Newtonian fluid. The measured values of whole blood from the micro-channel (4.17cP) and the vacuum method (4.22cP) at 500 s^−1^ were closely correlated at 27 °C. A calibration curve was established for viscosity as a function of hematocrits to predict a rise and fall in viscosity during embryonic development. Thus, our rapid capillary pressure-driven micro-channel revealed the Newtonian fluid behavior of zebrafish blood at high shear rates and the dynamic viscosity during development.

## Introduction

Blood viscosity is influenced by the ratio of the volume of red blood cells to the volume of whole blood or known as hematocrit. In the clinical setting, newborns with cyanotic congenital heart disease or adults with hematological diseases develop disorders in their hematocrit^1, 2^. In developmental biology, blood viscosity modulates fluid shear stress to regulate mechano-signal transduction during cardiac morphogenesis^3, 4^. However, acquiring accurate measurements of blood viscosity in developing embryos remains experimentally challenging. Furthermore, a high-throughput methodology to interrogate small-scale blood viscosity has not previously been performed.

To measure small-scale blood viscosity, we chose the zebrafish model for their high fecundity and short life cycle. Zebrafish provide a genetically tractable animal model system for organogenesis, disease, and drug discovery^5–7^. Embryonic zebrafish hearts develop contractile function within 120 hours post-fertilization, allowing for light-sheet microscopy and hemodynamic interrogation of cardiac development^3, 8^. Additionally, adult zebrafish have the capacity to regenerate their injured hearts to restore contractile function at 60 days post apical ventricular amputation^9, 10^. Evaluating the hemodynamics in these developmental and disease model contexts requires an accurate measurement of the blood viscosity, and thus interrogating the dynamics of blood viscosity during these developmental or disease stages is fundamentally significant^11^.

The minute amount of adult zebrafish blood (~20 μL) hampers viscosity measurement^12^. Conventional viscometers, including the cone-and-plate mechanism, require a large sample volume and prolonged measurement^13^. The scanning capillary-tube viscometer, mass detecting capillary viscometer, and pressure-scanning capillary viscometer provide rapid measurements (<3 min) without anti-coagulants^14–16^. Unfortunately, a large sample of blood volume (~3 mL) is required, rendering these devices less desirable for point-of-care applications and incompatible with measuring the blood viscosity of individual zebrafish. Using a pressure sensor with a syringe pump provides an alternative methodology to measure small amount of fluid^17^. However, the tiny amount of zebrafish blood coagulates rapidly during experimental set-up. For this reason, most investigators empirically assume similar rheological components in both zebrafish and humans, and most estimate the zebrafish blood viscosity on the basis of area or volume fraction^18–20^. At a given hematocrit count, previous studies have demonstrated variations in blood viscosity from different species^18^, Thus, the above assumption may be deemed inaccurate.

**Figure 1:**
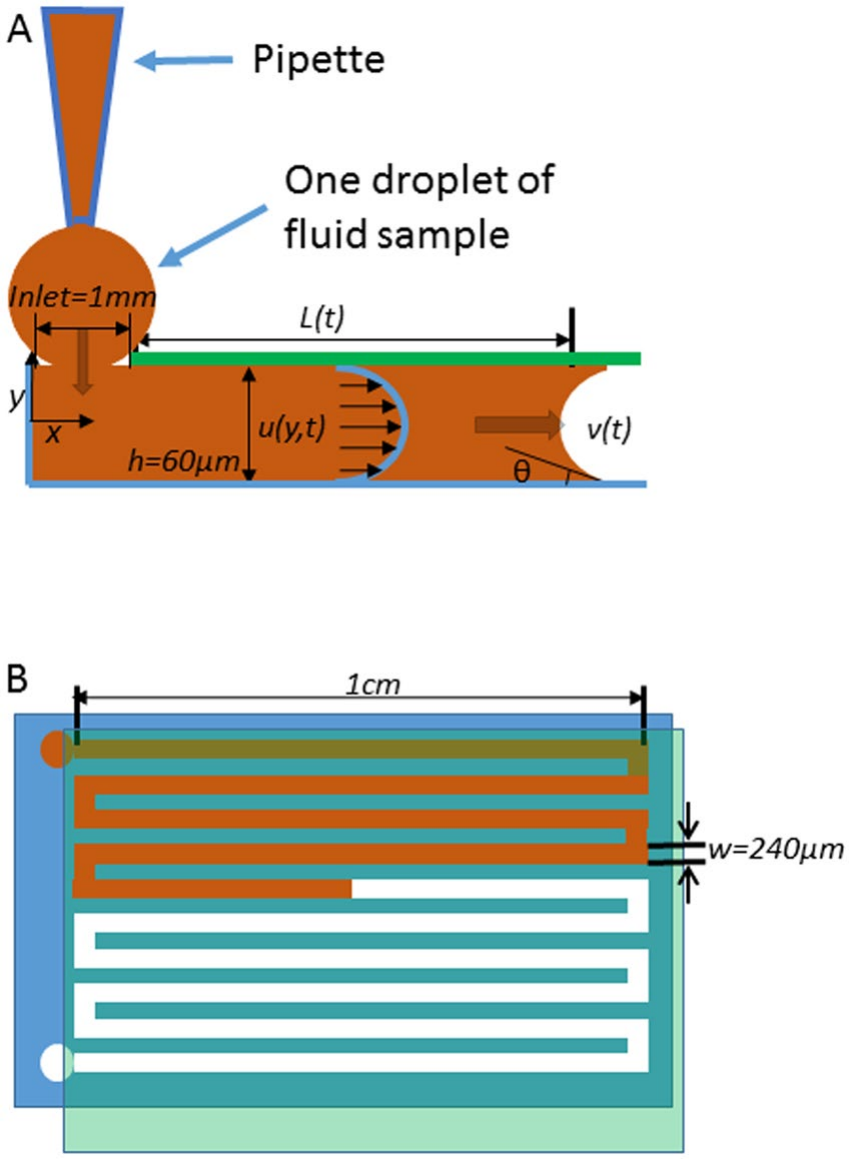
Capillary pressure-driven micro-channel. (**A**) Side view of the application of the fluid sample (beige color) into the inlet port. The sample was pipetted to the inlet of the channel. By virtue of the capillary force, the microfluidic device imbibed the fluid sample immediately. Velocity distribution *u*(*y,t*), channel height (*h*), contact angle (θ), and fluid traveling distance (*L*) are highlighted and labeled. (**B**) Top view of the microfluidic device, illustrating a blood sample being applied to the inlet port at the top left corner, and traveling through the micro-channel. The channel was bonded with Parylene-coated glass after oxygen plasma treatment.

In this context, we applied the capillary pressure-driven principle to develop microfluidic channels for the small-scale blood viscosity to achieve high-throughput measurement (Fig. 1). Unlike other viscometers, the microfluidic design allows for a minute sample volume (<3 μL) for rapid (<2 min) and continuous blood viscosity measurement over a wide range of shear rates. The experimentally acquired blood viscosity of water, blood plasma, and whole blood was further validated with a vacuum-driven parylene tube at high shear rates, at which point the zebrafish blood behaved as a Newtonian fluid. We further established a calibration curve for zebrafish blood viscosity to predict the transient rise and fall of viscosity during embryonic development. Thus, our capillary pressure-driven microfluidic platform provides a fundamental basis to rapidly measure zebrafish blood viscosity, to reveal the Newtonian fluid behavior at high shear rates, and to demonstrate the dynamic blood viscosity during development.

## Results

### Measurement of water viscosity for calibration of microfluidic channel

**Figure 2:**
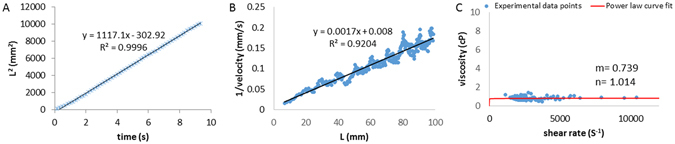
Calibration of water by the micro-channel at 27 °C. (**A**) The square of travel distance versus time scatter plot (asterisks) reveals the linear fitting based on the Newtonian fluid assumption (solid line, eq. (11) with n = 1). (**B**) The inverse of mean velocity versus travel distance scatter plot (square dots) supports the linear fitting based on the Newtonian fluid assumption (solid line, eq. (10) with n = 1). (**C**) Viscosity versus shear rates scatter plot (round dots) demonstrate the fitted power law parameters m and n in eq. (1). The method was adopted from Kang *et al*.^32^.

Water was used to calibrate the pressure-driven microfluidic channels. Furthermore, the individual micro-channels were calibrated against water to minimize fabrication error. The square displacement *(L* ^*2*^ *)* of water versus travel time demonstrates the Newtonian flow behavior of water (Fig. 2A). In addition, the inverse of mean velocity vs. travel distance of water reveals the Newtonian behavior (Fig. 2B). For a Newtonian fluid, the power law exponent (*n*) is commonly considered to be 1^21^. The power law exponent of water from our micro-channel device was 1.059 (Figure S1A), and the geometry factor was calibrated by the known water viscosity of 0.85 cP at 27 °C (Fig. 2C).

### Adult zebrafish blood viscosity

**Figure 3:**
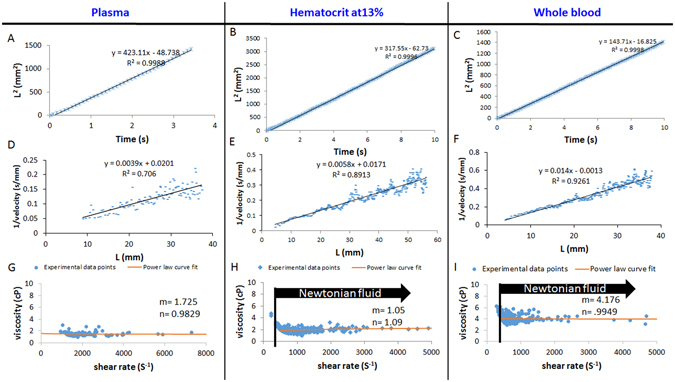
Alteration of zebrafish hematocrit in the capillary-pressure driven micro-channel at 27 °C. (**A**–**C**) The square of travel distance versus time scatter plot (asterisks) reveals the linear fitting based on the Newtonian fluid assumption (solid line, eq. (11) with n = 1). A = plasma, B = 13% hematocrit, C = whole blood. (**D**–**F**) The inverse of mean velocity versus travel distance scatter plot (square dots) supports the linear fitting based on the Newtonian fluid assumption (solid line, eq. (10) with n = 1). D = plasma, E = 13% haematocrit, F = whole blood. (**G**–**I**) Viscosity versus shear rate scatter plot (round dots) supports the fitted power law parameters m and n in eq. (1). The method was adopted from Kang *et al*.^32^. G = plasma, H = 13% haematocrit, I = whole blood.

After calibrating the micro-channel, we varied the zebrafish hematocrits at a given volume of 3 μL, and we applied zebraflish blood samples with different hematocrits to the micro-channels (Video S1). The square of travel displacement *(L* ^*2*^ *)* of blood vs. time demonstrates linear fitting consistent with a Newtonian model (Fig. 3A–C). Increasing the sample hematocrit reduced the velocity through the micro-channel in relation to variation in travel distance^22^. Scatter plots of the inverse of mean velocity vs. travel distance demonstrates linear fitting based on the Newtonian fluid assumption (Fig. 3D–F)^23^. Power-law exponents were obtained by log [1/L] vs. log [velocity] (Figure S1B–D). The viscosity of blood plasma was fairly consistent at 1.5 cP over a range of shear rates (Fig. 3G). Increasing the hematocrit elevated the zebrafish blood viscosity. The viscosity of both hematocrit at 13% and the whole blood also behaved as a Newtonian fluid at high shear rates > 500 s^−1^ (Fig. 3H,I). Zebrafish blood was well-fitted in the two-parameters (m,n) power-law fluid model as performed by MATLAB (MathWorks, Natick, MA) power-law curve fitting app.

### Validations in viscosity

**Figure 4:**
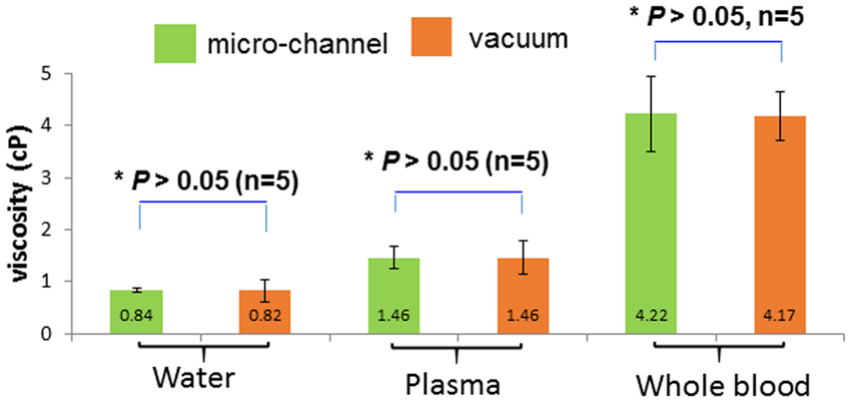
Comparison and validation of zebrafish blood viscosity measurements. The values of viscosity acquired by the vacuum-driven Parylene tubes demonstrated good agreement with those obtained by the pressure-driven microfluidic channels (shear rate > 500 s^−1^). Both measurements were at 27 °C.

To validate the pressure-driven micro-channel, we induced vacuum pressure (−0.9 bar) to micro-fabricated Parylene-based tubes (inner diameter = 400 μm). The contact angle with the channel was maintained at 90° to minimize the capillary pressure from interrupting the vacuum pressure during vacuum suction. The viscosities of water, plasma, and whole blood were compared. A negative pressure of −0.9 bar was applied to the Parylene-based tubes that were filled with heparinized zebrafish blood and plasma, respectively (Figure S2). This Parylene-based tubes reproduced and validated the viscosity values measured by our micro-channels (*p > 0.05 n = 5). Thus, the values of our vacuum-driven methodology were in agreement with those obtained by the capillary pressure-driven micro-channeld (Fig. 4 and Video S2).

### Embryonic zebrafish blood viscosity

**Figure 5:**
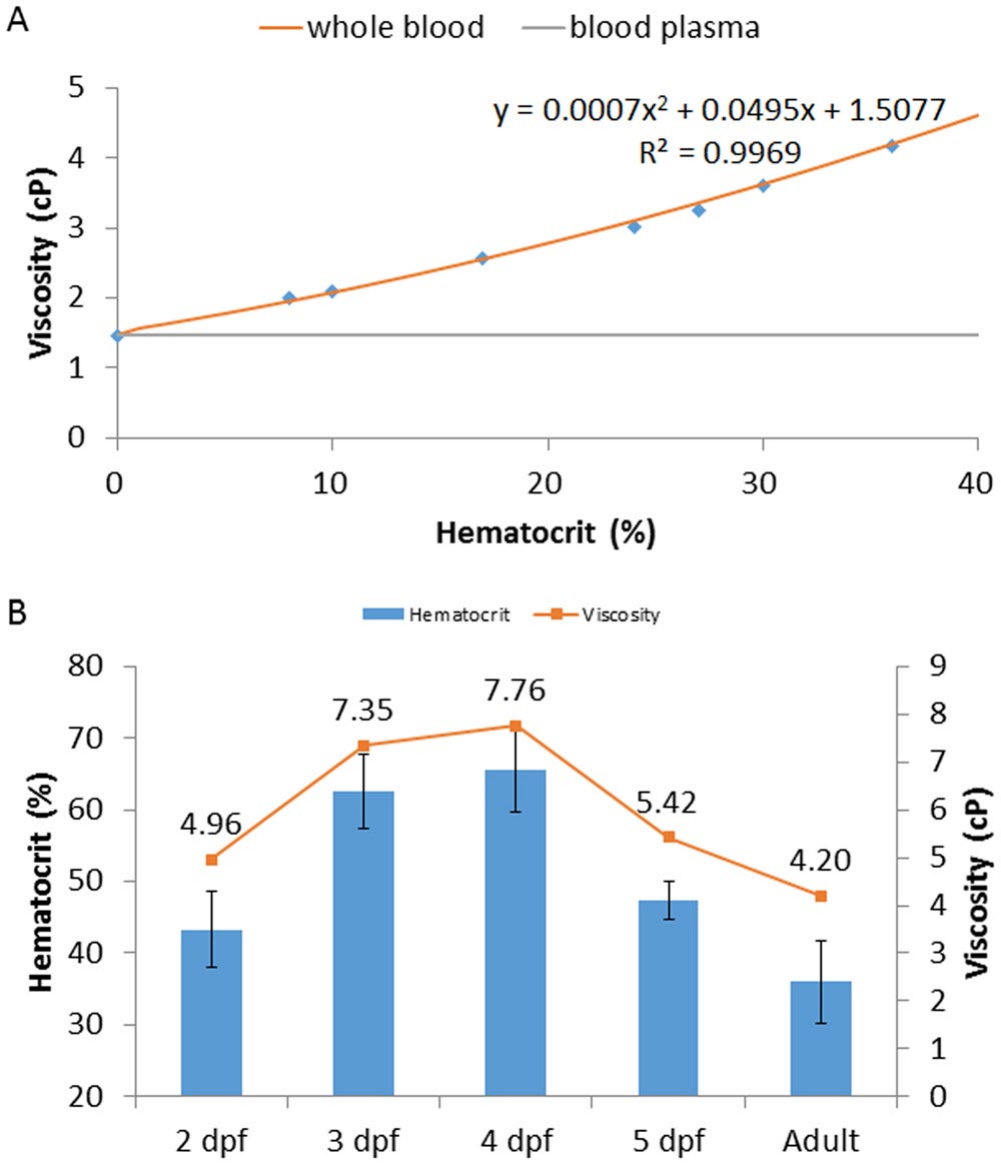
Calibration curves for Zebrafish blood viscosity as a function of hematocrits. (**A**) A second order polynomial curve was in close agreement with the experimental values of blood viscosity. (**B**) The calibration curves in (**A**) predicted the dynamic rise and fall in embryonic zebrafish blood viscosity from 2 dpf to 5 dpf to adult fish. The labelled values are those of the predicted blood viscosities.

A second order polynomial curve, which is currently used to plot human hematocrit versus viscosity and fit the most reliable (Figure S3), was derived from the experimentally acquired adult zebrafish blood viscosity over a range of hematocrits (Fig. 5A)^24^. This curve provides the basis to extrapolate the viscosity of embryonic zebrafish. We measured the hematocrits starting at 2 days post fertilization (dpf) to 5 dpf, during which cardiac looping and valvular formation occurred^4^. We discovered an increase in viscosity from 2 dpf to 4 dpf, followed by a decrease from 5 dpf to adult stage when the hematocrit decreased from 47% to 36% (Fig. 5B). In line with Fig. 3D, zebrafish blood plasma behaved as a Newtonian fluid at 1.5 cP.

## Discussion

The contribution of our study resides in 1) demonstrating the Newtonian fluid behavior of zebrafish blood at shear rates >500 s^−1^, and 2) predicting the dynamics of blood viscosity during embryonic development (Fig. 5B). Our pressure-driven micro-channels addressed the experimental challenge of micro-scale sample volume. The operational principle and channel configuration were compared with the vacuum-driven Parylene tube system to validate the viscosity values in water, blood plasma, and whole blood (Fig. 4)^20^. The power law exponent of zebrafish blood remained close to 1 over a range of hematocrits consistent with the Newtonian fluid behavior^23^. Zebrafish blood develops Newtonian behavior at high shear rates > 500 s^−1^, whereas human blood starts to develop Newtonian behavior at lower shear rates > 200 s^−1^ ^22^. Both human and zebrafish blood develops non-Newtonian behavior when viscosity increase exponentially with decreasing shear^22^. We further established a calibration curve for zebrafish blood viscosity, from which we provided the first observation for the dynamic rise and fall of viscosity during cardiac looping and valvular formation.

Capillary pressure-driven channels allow for direct interrogation of blood viscosity from the small-scale volume as opposed to the use of area or volume fraction rate of blood particles^12, 13, 21^. In addition, microfluidic channel allows for rapid viscosity measurement, whereas vacuum chamber requires a long time interval for sample transfer. Previous representative zebrafish blood viscosity was inadequate to study blood rheology at low shear rate < 500 s^−1^. Our experimentally derived calibration curve (Fig. 5A) provides a dynamic range of zebrafish blood viscosities as a function of hematocrit as a basis to investigate fluid shear stress modulation of developmental gene expression and cardiac development^3, 19^.

We demonstrated the dynamics of zebrafish blood viscosity during embryonic development initially increasing until 4 dpf and starting to decrease until adult stage. In the early embryonic stage, zebrafish develop a tube-like structure undergoing peristaltic contraction^20^. Despite literature search, the transient rise and fall of embryonic blood viscosity remains unknown. During development, the peristaltic heart tube undergoes cardiac looping at ~20 hours post-fertilzation (hpf), followed by atrio-ventricular (AV) valvular formation from 80 hpf to 120 hpf. These structural changes occur in parallel with the rise and fall of blood viscosity. Using the empirically-derived blood viscosity, we previously demonstrated computational fluid dynamics-derived average peak shear stress increases from 4.0 ± 0.1dynes·cm^−2^ at 20 hpf to 29 ± 8 dynes·cm^−2^ at 60 hpf to 81 ± 11dynes·cm^−2^ at 120 hpf. Due to red blood cell deformation and lysis during centrifugation, we did not measure blood with high hematocrit^25, 26^. Our current capillary pressure-based micro-channel further provides an experimental basis to determine the temporal variations in shear stress during cardiac development.

Fluid shear stress is intimately linked with valvular formation. Reducing viscosity due to flow regurgitation across the AV valves was implicated in valvular anomalies in the zebrafish embryos^19^. Reducing viscosity by *gata1a* morpholino oligonucleotide injection reduced endocardial Notch signaling to attenuate trabeculation in the zebrafish embryos, whereas increasing viscosity by erythropoietin mRNA injection elevated shear stress to promote endocardial ridge-like formation for trabeculation^3^. In addition, shear stress activated endothelin-1 (ET-1), lung Krüppel-like factor (KLF-2) and endothelial nitric oxide synthase (eNOS) expression in the chicken embryonic model of cardiac development^27^. Shear stress further activated Wnt-Angiopoietin-2 signaling to restore vascular repair in zebrafish embryos^28^. Thus, direct measurement of viscosity provides a rheological basis to elucidate developmental biomechanics and physiology during development.

The advent of a MEMS-based viscometer has enabled a micro-scale of sample volume^29^. This MEMS-based oscillating micromechanical viscometer measures zebrafish viscosity at a specific shear rate at one time point, rendering the process laborious and prolonged when the viscosity values for a wide range of shear rates are desired. Our pressure-driven micro-channel allows for small sample volume, rapid acquisition, and reproducible viscosity values as validated by the vacuum-driven Parylene tubes. The limitation of the current study lies in the need for the heparin-coated capillary channel to prevent acute thrombosis due to 1) rapid blood coagulation and 2) pooling plasma from different zebrafish. Shear thickening occurred by altering hematocrit in the blood sample with power law exponent *n* > 1. For this reason, blood coagulation, or thrombosis, developed when the whole blood was pooled from different fish. Moreover, we have ignored the entrance effect due to small size (*h* = 60 *μm*, *w* = 240 *μm*) of the micro-channel. This entrance effect becomes insignificant beyond a channel length of 10 times the cross length of channel^30^.

Overall, we demonstrate the Newtonian fluid behavior of zebrafish blood, and we established the calibration curve for zebrafish viscosity as a function of hematocrit to predict the dynamics of blood viscosity during development. Thus, direct measurement of the blood viscosity from the zebrafish provides the rheological basis to study developmental biomechanics with a translational implication for point-of-care measurements for the new-born.

## Methods

### Maintaining of Embryonic Zebrafish

Wild type zebrafish and double transgenic line, *Tg(fli1a:GFP; gata1:DsRed)*, were bred and maintained at the UCLA zebrafish core facility. All zebrafish experimentation in this study was carried out in accordance with animal protocols approved by UCLA Institutional Animal Care and Use Committee (IACUC) protocols (ARC#: 2015-055 and 2012-039). Zebrafish aquarium was maintained at 27~28 °C for the experiments.

### Collection of Zebrafish Blood

Adult zebrafish blood was collected after euthanization with tricaine methane sulfonate (MS-222). A lint-free paper tower (TexWipe, Kimberly-Clark, Irving, TX) was used to remove the water from zebrafish body surface. A small incision to the chest was performed with Vannas scissors (FST, CA) to expose the hearts. A lint-free paper tower was used again to remove the fluid from the incised chest surface. After zebrafish heart incision, heparinized microhematocrit tubes (1-000-3200-H, Drummond Scientific, PA) were used to collect blood from the hearts by capillary forces. This procedure was performed at 27 °C to maintain aquatic temperature at which the zebrafish were bred and raised.

### Alteration of hematocrits

After blood collection, one end of the microhematocrit tubes was sealed by the hematocrit sealer (Critoseal^TM^, Leica Biosystem, IL). The sealing was accomplished by cutting 20 μL pipette tips (Microloader^TM^, Eppendorf), followed by dipping these tips onto the hematocrit sealer as a cap. To dilute the blood, we first prepared the blood plasma by centrifuging one tube of whole blood sample at 10 krpm (~9000 × G) for 1 minute (Minispin^TM^, Eppendorf). We assumed that the most of components affecting coagulation were centrifuged and precipitated. Next, the plasma was fully mixed with another tube of whole blood sample to obtain the diluted blood. Afterwards, half of the diluted blood sample was added to the microfluidic channel to measure the viscosity and the other half was centrifuged again to determine the hematocrit (Figure S4). The hematocrit was successfully lowered to 13% and 24% in two separate measurements. Thus, we were able to establish hematocrit values between 0% (blood plasma) and 35% (whole blood).

### Theoretical calculation of shear rate and viscosity

The majority of shear thinning liquids, including blood, were modeled by using a two-parameter power law fluid model, also known as the Ostwalt-de Waele relationship:

1 $$\mu =m{\dot{\gamma }}^{n-1}\,$$

2 $$\tau =\mu \dot{\gamma }=m{\dot{\gamma }}^{n}\,$$

where *m* and *n* are parameters pertaining to the fluid characteristics, *μ* is the viscosity, $\dot{\gamma }$ is the shear rate, and *τ* is the shear stress^22, 31^. The Navier-Stokes equation at quasi-steady state is simplified for a quasi-1-dimensional Hagen-Poiseuille flow in the thin rectangular channel (Fig. 1)^32–34^.

3 $$0=-\,\frac{dp}{dx}+\frac{\partial \tau }{\partial y}=\frac{{\rm{\Delta }}p}{L(t)}+\frac{\partial (m{\dot{\gamma }}^{n})}{\partial y}$$

4 $${\rm{\Delta }}p=2\sigma (\frac{1}{h}+\frac{1}{w})\cos \,\theta \,$$

5 $$\tau =\tau (y,t),\,\dot{\gamma }=\dot{\gamma }(y,t)$$

where *τ* denotes shear stress, Δ*p* the Young-Laplace pressure drop, *L*(*t*) the travel distance in the channel, *σ* the surface tension, *θ* the contact angle, *h* the channel height, *w* the channel width and $\dot{\gamma }$ the shear rate.

The wall shear rate is related to the mean flow velocity$\,v(t)$ as follows^35^.

6 $${\dot{\gamma }}_{w}(t)=\dot{\gamma }(\frac{h}{2},t)=\frac{Sv(t)}{2h}\frac{2n+1}{3n}$$

7 $$v(t)=\frac{dL(t)}{dt}$$

where *S* is a constant depending on the channel geometry^36^. By (1) solving the Navier-Stokes equation at quasi-steady state, and (2) applying the wall shear rate to the power-law model, the following two expressions for the wall shear stress are obtained:

8 $${\tau }_{w}(t)=-\tau (\frac{h}{2},t)=\frac{h{\rm{\Delta }}p}{2L(t)}$$

9 $${\tau }_{w}(t)=m{[{\dot{\gamma }}_{w}(t)]}^{n}=m{[\frac{2n+1}{6nh}Sv(t)]}^{n}$$

Therefore, the relationship between the travel distance *L*(*t*) and mean flow velocity *v*(*t*) is expressed as follows^13^:

10 $$\frac{1}{L(t)}=\frac{2m}{{h}^{n+1}{\rm{\Delta }}p}{(\frac{2n+1}{6n}S)}^{n}v{(t)}^{n}$$

Solving *L*(*t*) as a function of time with the initial condition, *L*(0) = 0, the result is:

11 $$L(t)=h{(\frac{{\rm{\Delta }}p}{2m})}^{\frac{1}{n+1}}{(\frac{6n+6}{2n+1}\frac{t}{S})}^{\frac{n}{n+1}}$$

In addition, the mean flow velocity and fluid viscosity can be derived as:

12 $$v(t)=\frac{dL(t)}{dt}=nh{(\frac{6}{2n+1}\frac{1}{S})}^{\frac{n}{n+1}}{(\frac{{\rm{\Delta }}p}{2n+2}\frac{1}{mt})}^{\frac{1}{n+1}}$$

13 $$\mu (t)=m{[{\dot{\gamma }}_{w}(t)]}^{n-1}={m}^{\frac{2}{n+1}}{(\frac{2n+1}{12n+12}\frac{S{\rm{\Delta }}p}{t})}^{\frac{n-1}{n+1}}$$

### Calibration against water and estimation of the channel geometry factor (*S*)

During the calibration *L*(*t*) was video recorded at 30 fps (EOS 7D, Canon, Tokyo, Japan) and later tracked frame-by-frame using ImageJ (Figure S4). The contact angle *θ* was averaged from all frames (Figure S5). Contact angles were manually measure by ImageJ (NIH, Bethesda, MD). The channel height *h* and channel width *w* were measured using the surface profiler (P-15, KLA-Tencor). To estimate the channel geometry factor (*S*), water was chosen as the calibrating fluid given the well-defined power-law parameters *m*, *n*, and surface tension (*m* = 0.851cP, *n* = 1, *σ* = 71.8 mN/m at 27 °C). Then S was estimated according to eq. (11). We placed PDMS-based micro-channel on the hot plate to pre-heat (27 °C) before experiments.

### Measuring the power-law parameters of blood

The same experimental calibration was applied to measure the blood viscosity. The surface tension *σ* of whole blood and water at 27 °C is 0.058 N/m and 0.072 N/m, respectively^34^. We also assumed that the surface tension blood plasma and whole blood was the similar^34, 37^. Finally, the power-law parameters, *m*, *n* were found by fitting the equation of *L*(*t*) by assuming the same channel geometry factor *S* from the water calibration.

### Embryonic zebrafish hematocrit

The double transgenic zebrafish line, *Tg(fli1a:GFP;gata1:DsRed)*, was bred at the UCLA zebrafish core facility. From 2 dpf to 5 dpf, we mounted fish to an inverted microscope (IX71, Olympus, Tokyo, Japan). GFP and DsRed images of the tail region were captured by camera (EOS 7D, Canon, Tokyo, Japan) and merged using ImageJ (NIH, Bethesda, MD). Using area fraction analysis, we were able to derive the hematocrit counts at various developmental stages of zebrafish embryos.

### Fabrication of microfluidic channel

The microfluidic channel was patterned on a silicon wafer by the DRIE process and then molded by PDMS (MED-6219, NuSil Technology). After releasing from the wafer, the PDMS mold and a Parylene-coated glass slide were made hydrophilic by oxygen plasma treatment with 50 Watt power and 200 mTorr O_2_ pressure for 1 minute, and bonded together to create the rectangular channel. The entire device takes up an area about 1 cm^2^, with channel height (*h*) = 60 μm and width (*w*) = 240 μm (Fig. 1). Despite the well-recognized surface roughness of PDMS topography at the nano-scale, this effect on thrombosis was experimentally insignificant at the micro-scale.

### Vacuum-driven capillary viscometer

A glass capillary tube (1‐000‐0050, Drummond Scientific, O.D. = 940 μm, I.D. = 447 μm) was uniformly coated with Parylene-N (thickness = 2 μm), a transparent and biocompatible polymer, by chemical vapor deposition (PDS 2010, SCS) at room temperature to maintain a ~90° water contact angle and minimize the capillary pressure. While one end of the tube acted as the inlet for blood inflow, the other end was connected to a vacuum chamber to generate a pneumatic pressure difference Δ*p* = 0.9 bar (Figure S2). The viscosity of blood *μ*was calculated from the Hagen-Poiseuille equation:

14 $${\rm{\Delta }}p=\mu \frac{32L(t)v(t)}{{d}^{2}}$$

where *d* is the tube diameter, *L*(*t*) the length of the blood column and *v*(*t*) the mean flow velocity. The shear stress and shear rate at the inner tube wall are as follows:

15 $$\,{\tau }_{w}=\frac{d{\rm{\Delta }}p}{4L(t)},\,{\dot{\gamma }}_{w}=\frac{8v(t)}{d}$$

## Electronic supplementary material

Video S1

Video S2

Supplementary Materials
