# Supplementary material for: A Rapid Capillary-Pressure Driven Micro-Channel to Demonstrate Newtonian Fluid Behavior of Zebrafish Blood at High Shear Rates
Source: Sci Rep. 2017 May 16;7:1980. doi: 10.1038/s41598-017-02253-7 (PMC5434032; doi:10.1038/s41598-017-02253-7)
Supplement: Supplementary file 3 — Supplementary Materials [file 41598_2017_2253_MOESM3_ESM.pdf]

Supplementary Information

A Rapid Capillary-Pressure Driven Micro-Channel to Demonstrate Newtonian Fluid Behavior of Zebrafish Blood at High Shear Rates

Juhyun Lee[1,7], Tzu-Chieh Chou[2,7], Dongyang Kang[2], Hanul Kang[3], Junjie Chen[1], Kyung In Baek[1,7], Wei Wang[4], Yichen Ding[1], Dino Di Carlo[1,5], Yu-Chong Tai[2], Tzung K. Hsiai[1,5,6]

[1]Department of Bioengineering, University of California Los Angeles, Los Angeles, CA, 90095, USA
[2]Department of Electrical Engineering, California Institute of Technology, Pasadena, CA, 91125, USA
[3]Division of Cardiology, Veterans Affairs Greater Los Angeles Healthcare System, Los Angeles, California 90073, USA
[4]Department of Electrical Engineering, Peking University, Beijing, 100871, China [5]California NanoSystem Institute, University of California Los Angeles, Los Angeles, CA, 90095 USA,
[6]Department of Medicine (Cardiology), School of Medicine, University of California Los Angeles, Los Angeles, CA, 90095, USA

[7]The authors contributed equally to this work

Corresponding Author:
Tzung K. Hsiai, MD, PhD,
Departments of Medicine and Bioengineering
University of California, Los Angeles (UCLA) Los Angeles, CA.
Email: THsiai@mednet.ucla.edu.
Phone: 310-268-3839.
Fax: 310-268-4288

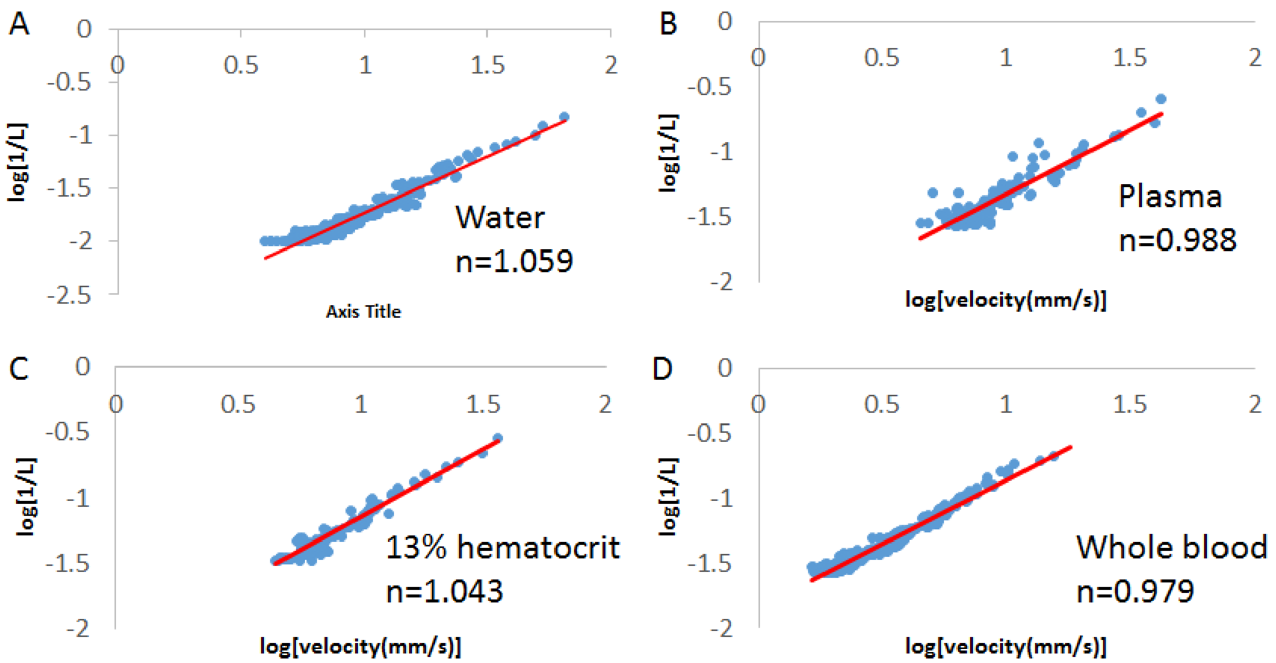

Figure S1. Demonstration of log [1/L] vs. log [velocity] plot. (A) Power law exponent of water as a function of the slope of log [1/L] vs. log [velocity] was 1.059. (B,C,D) Power law exponent of zebrafish blood plasma, 13% hematocrit, and whole blood as the slope of log [1/L] vs. log [velocity] was close to 1.

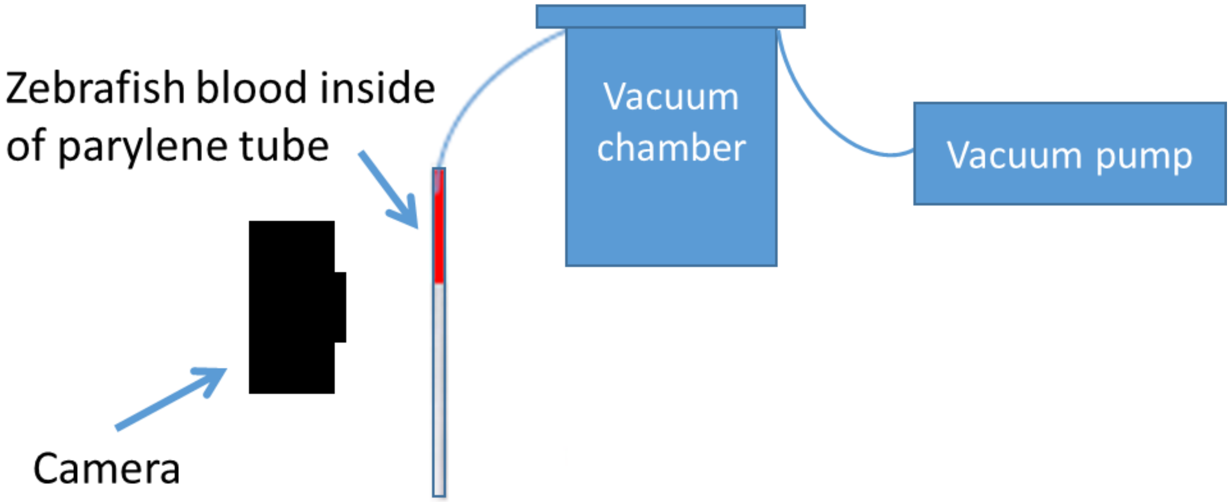

Figure S2. Viscosity measurements by the vacuum pump. The vacuum pump generated a negative pressure at -0.9 bar to the chamber that was connected to the parylene tube filled with zebrafish blood. Camera was used to capture the motion of fluid by the suction force.

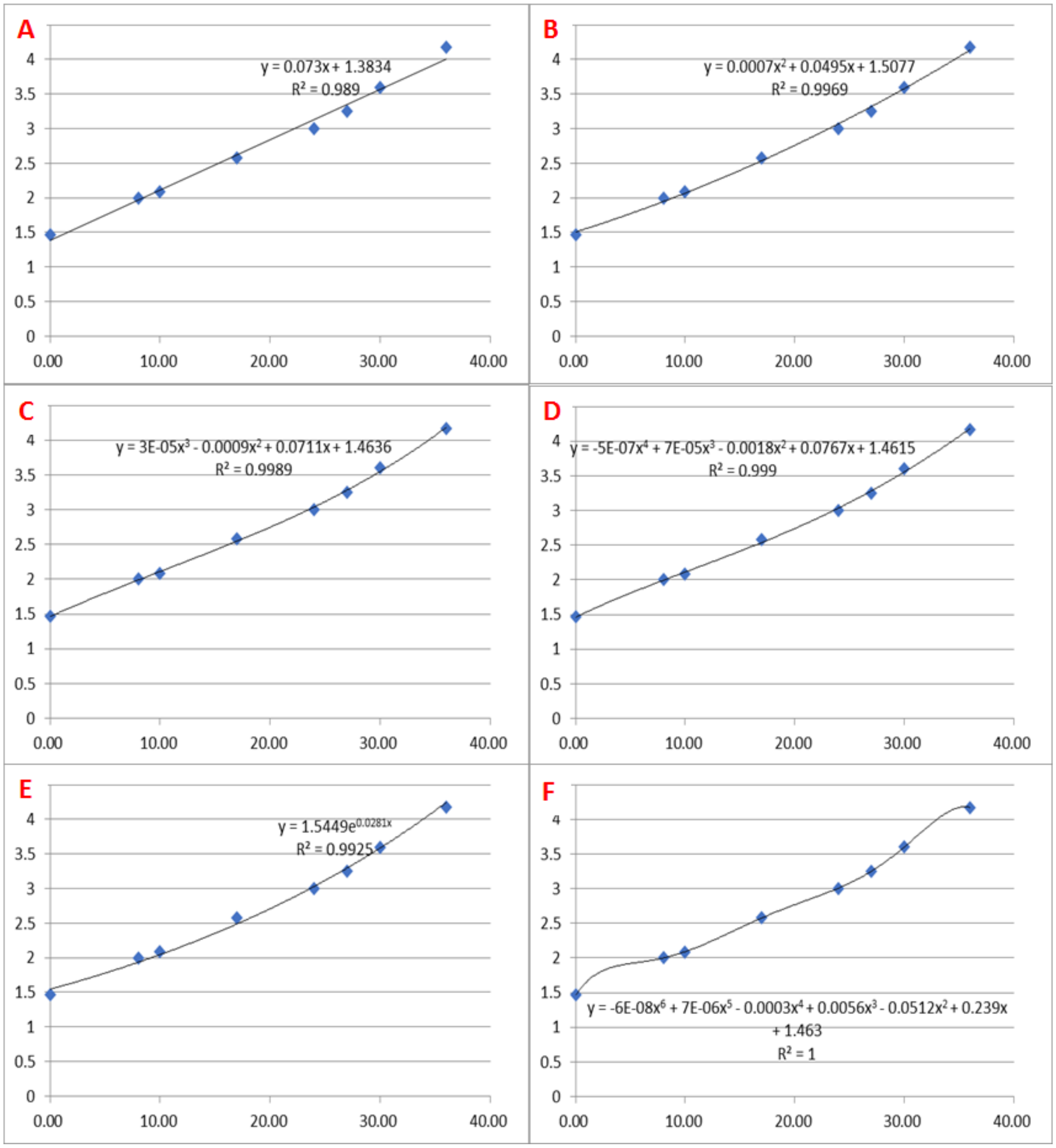

Figure S3. Multiple curve fitting for zebrafish blood viscosity. (A) First order polynomial curve fitting. (B) Second order polynomial curve fitting. (C) Third order polynomial curve fitting. (D) Fourth order polynomial curve fitting. (E) Exponential curve fitting. (F) Sixth order polynomial curve fitting.

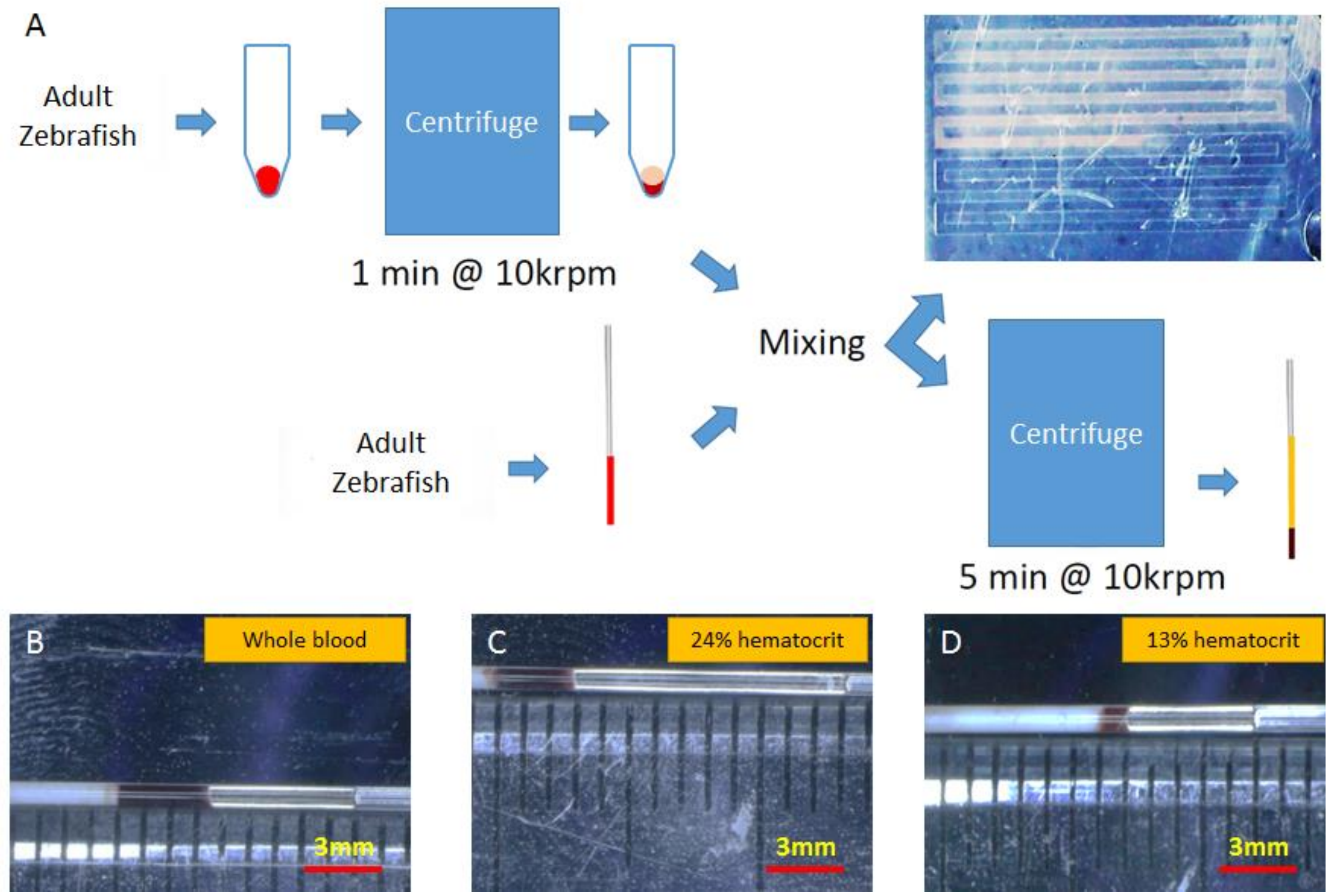

Figure S4. Alteration of zebrafish blood hematocrit. (A) Collected zebrafish blood was immediately transferred to a 1.5mL tube for centrifuge at 10 krpm for 1 minute to minimize blood coagulation. In parallel, heparinized glass capillary tubes were used to collect the whole blood. After centrifuge, the blood plasma and red blood cells were clearly separated. New tubes were used to mix the whole blood with the blood plasma. This blood was used to apply to the micrco-channels and to determine the hematocrit. (B,C,D) Altered hematocrit was established.

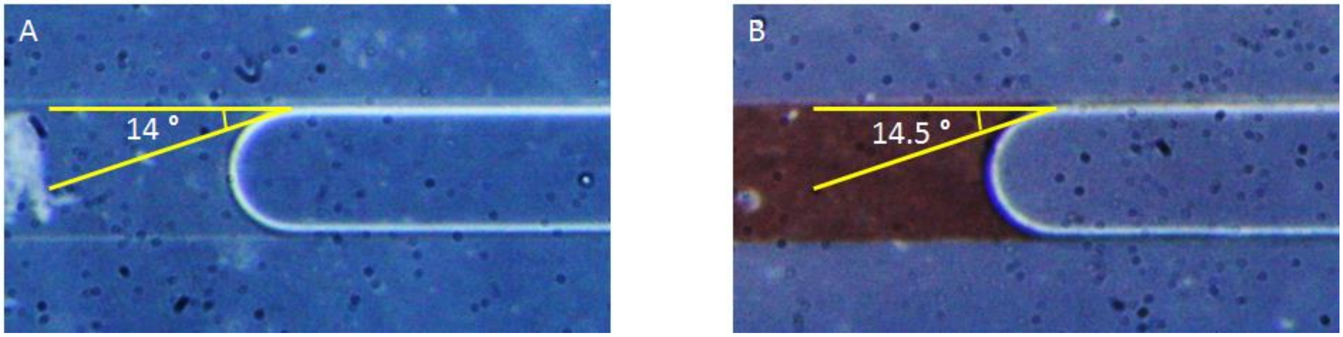

Figure S5. (A) Contact angles of blood plasma and (B) zebrafish whole blood.

Video S1 shows fluid flow after zebrafish blood was applied into microfluidic channel.

Video S2 shows fluid flow after zebrafish blood was applied into vacuum chamber.
